# Supplementary material for: Bio-Inspired Fiber Reinforcement for Aortic Valves: Scaffold Production Process and Characterization
Source: Bioengineering (Basel). 2023 Sep 9;10(9):1064. doi: 10.3390/bioengineering10091064 (PMC10525898; doi:10.3390/bioengineering10091064)
Supplement: Supplementary file 1 [file bioengineering-10-01064-s001.zip › bioengineering-2584935-supplementary.pdf]

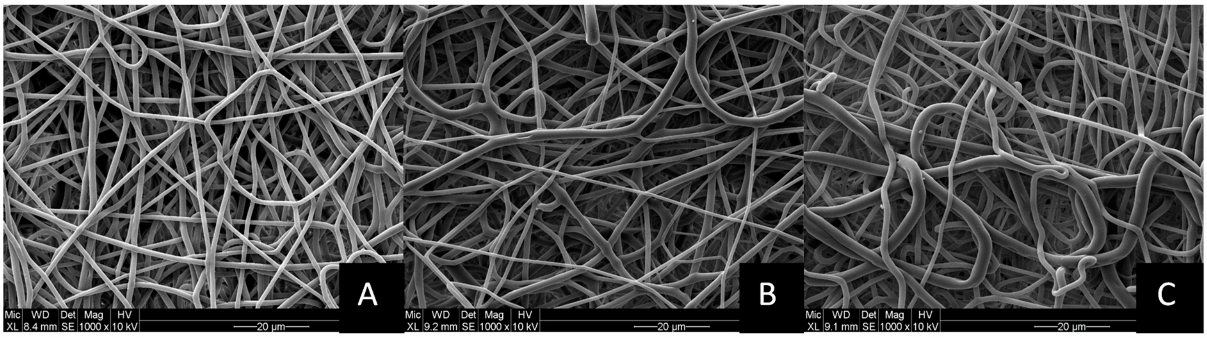

Sup. Figure S1. Effect of voltage on the e-spun fiber mat PCU concentration: 8 wt %; Distance to collector: 10 cm; A: 24 kV Fiber diameter 1.17 ± 0.49, pore size 5.31 ± 0.10; B: 26 kV fiber diameter 1.41 ± 0.54, pore size: 4.64 ± 0.48; C: 28 kV fiber diameter 1.62 ± 0.60, pore size: 4.73 ± 0.29; Fiber diameter and pore size are measured in [um]

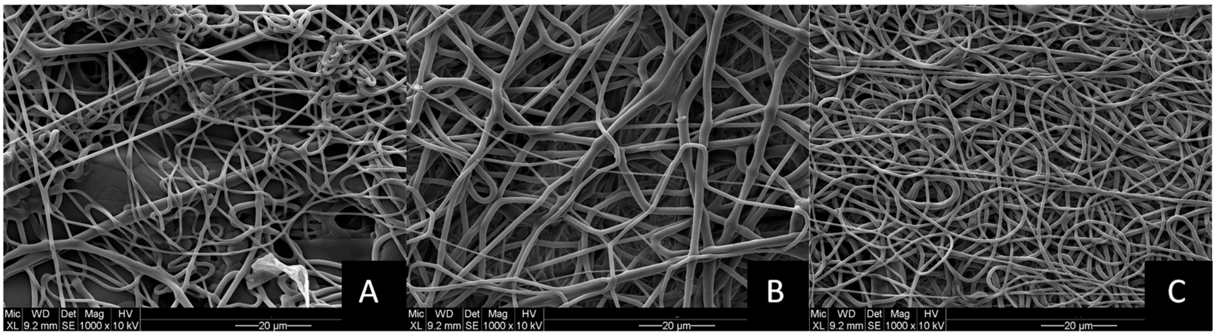

Sup. Figure S2. Effect of distance to collector on the e-spun fiber mat PCU concentration: 8 wt %; Voltage: 26 kV; A: 5 cm, Fiber diameter 1.76 ± 0.85, pore size 4.07 ± 0.59; B: 10 cm, fiber diameter 1.41 ± 0.54, pore size: 4.64 ± 0.48; C: 15 cm, fiber diameter 0.99 ± 0.32, pore size: 3.48 ± 0.24; Fiber diameter and pore size are measured in [um]

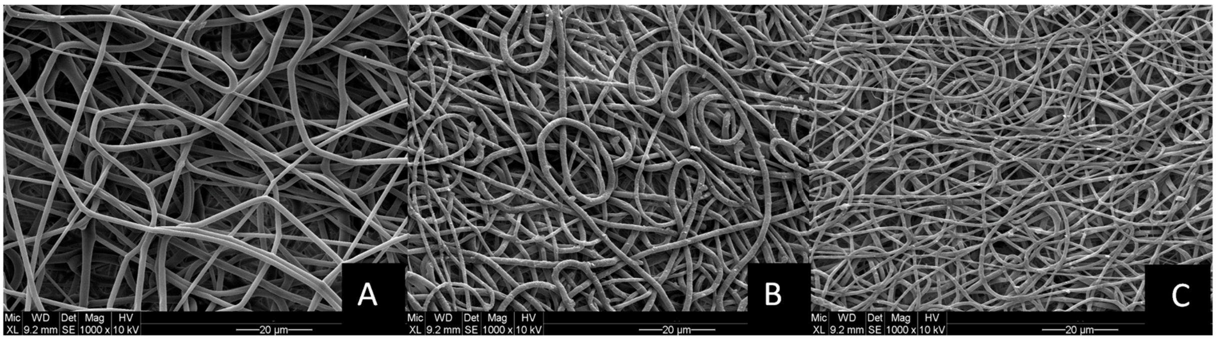

Sup. Figure S3. Effect of PCU concentration on the e-spun fiber mat distance to collector: 10 cm; Voltage: 26 kV; A: 8 wt%, Fiber diameter 1.50± 0.44, pore size 4.50 ± 0.37; B: 10 wt %, fiber diameter 1.47 ± 0.55, pore size: 3.86 ± 0.51; C: 12 wt%, fiber diameter 0.97± 0.35, pore size: 3.15 ± 0.06; Fiber diameter and pore size are measured in [um]
